# Synchronous Choriocarcinoma and Epithelioid Trophoblastic Tumor Concurring at the Cesarean Scar: A Case Report and Review of the Literature

**DOI:** 10.1155/2019/5093938

**Published:** 2019-09-16

**Authors:** Chunyan Zeng, Shadi Rezai, Alexander C. Hughes, Cassandra E. Henderson, Juan Liu

**Affiliations:** ^1^Key Laboratory for Major Obstetric Diseases of Guangdong Province, Key Laboratory of Reproduction and Genetics of Guangdong Higher Education Institutes, Department of Obstetrics and Gynecology, The Third Affiliated Hospital of Guangzhou Medical University, Duobao Road 63, Liwan District, Guangzhou, Guangdong 510150, China; ^2^Division of Minimally Invasive Gynecologic Surgery, Department of Obstetrics and Gynecology, Baylor College of Medicine, 6651 Main Street, 10^th^ Floor, Houston, TX 77030, USA; ^3^St. George's University, School of Medicine, St. George's, Grenada; ^4^Maternal and Fetal Medicine, Department of Obstetrics and Gynecology, Lincoln Medical and Mental Health Center, 234 East 149^th^ Street, Bronx, NY 10451, USA

## Abstract

We present a complicated case of recurrence of gestational trophoblastic neoplasms (GTN), mixed ETT and choriocarcinoma at an abdominal cesarean scar. This tumor consisted of typical morphologic and immunophenotypic features of ETT and choriocarcinoma. The tumor recurred despite the patient undergoing chemotherapy. The patient had this abdominal mass resected three times. The elements of ETT and coexisting choriocarcinoma varied each time. Due to re-recurrence of the tumor, the following decisions had been made: total abdominal hysterectomy, bilateral salpingectomy, right-sided inguinal lymph node biopsy. At the time of this report, recurrence was negative.

## 1. Introduction

Gestational trophoblastic neoplasms (GTN) are a group of neoplasms from fetal trophoblastic cells including choriocarcinoma (CC), epithelioid trophoblastic tumors (ETT), and placental site trophoblastic tumors (PSTT) [1]. They mostly exist separately, but there have been reported cases of mixed GTN with a combination of histologic CC, ETT, or PSTT [1–8]. ETT is a lesion of chorionic-type intermediate trophoblast cells appearing as a discrete, hemorrhagic, solid, or cystic lesion [1]. As a contrast, CC is a malignant tumor without chorionic villi, abnormal syncytiotrophoblast and cytotrophoblast, necrosis, and hemorrhage [9]. These tumors may be found on the uterine fundus, lower uterine segment, endocervix, and the broad ligament [1].

We present a case of mixed ETT and choriocarcinoma in proximity to a well healed cesarean delivery scar. This case is rather rare. Surgery may be the best treatment for mixed GTN.

## 2. Case Report

A 39-year old previously healthy woman went to an external institute after noticing an abdominal mass in her cesarean scar in December 2014. She had undergone two previous cesarean deliveries in 2005 and 2011 and also a surgical abortion in 2010. She claimed that she had not any previous history of malignancy, family history of malignancy, or using tobacco either.

This woman received the first mass resection in early June 2015 at the external institute mentioned above. She was diagnosed as having malignant endometriosis in a nonuterine site, and having hyperplastic and atypical trophoblastic cells by histological examination. These cells resembled squamous cell nests with necrosis and hemorrhage. This patient was referred to our institute.

The patient firstly attended to our clinic at the end of June 2015. Via laboratory analysis, we understood that total-human chorionic gonadotropin (t-HCG), *α*-feto-protein, CA-125, and carcinoembryonic antigen (CEA) were at normal serum levels. Further investigations with computed tomography (CT) of the chest and pelvis, and magnetic resonance image (MRI) of her head suggested negative for metastases. A pathologist specializing in gynecological malignancies in our institute diagnosed her with CC and ETT after examining her abdominal mass excision specimen provided by the external institute. Microscopically, there were sections of medium-sized cells with discernible nucleolus arranged in nests. Moreover, the tumor cells had polygonal nuclei with moderate nuclear pleomorphism and were surrounded by extensive necrosis. Immunohistochemistry (IHC) showed a highly positive staining for cytokeratin (CK), epidermal growth factor receptor (EGFR), human placental lactogen (HPL), p40 and p63; diffusely positive staining for inhibin-alpha and calretinin, and focally positive for HCG. The Ki-67 index was expressed in 50% of the tumor cells.

The patient received endometrial curettage in late June 2015. Biopsy of the lesion showed proliferative endometrium. Then, the patients received etoposide 100 mg/m^2^ and cisplatin 20 mg/m^2^ on Days 1 through 5 of 21-day cycles and continued for five courses.

After finishing chemotherapy, the patient received follow-up measurements monthly with serum t-HCG level, abdominal ultrasound, pelvic ultrasound, and chest X-ray. On 7th Jan. 2017, ultrasound at external institute indicated two hypoechoic nodules of sizes 18 × 5 mm and 7 × 5 mm, respectively, in subcutaneous soft tissues close to abdominal cesarean scar and normal t-HCG level (0.592 IU/L).

**Figure 1:**
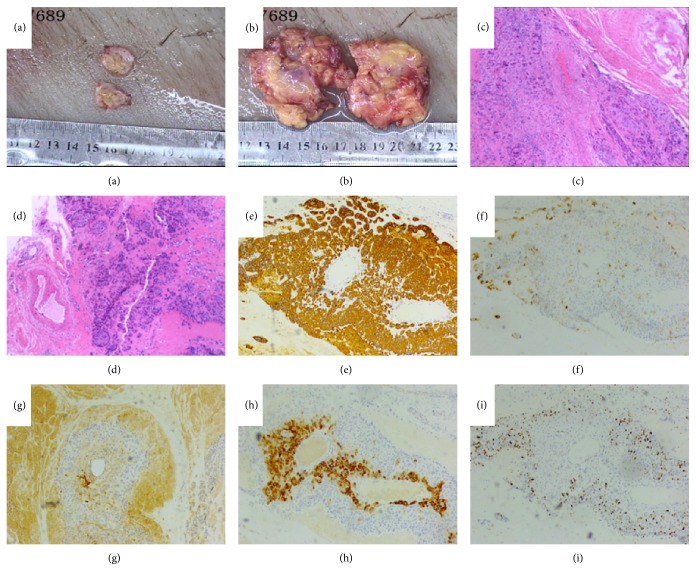
Macroscopical and microscopical features of her second time abdominal wall mass resection. (a and b) Abdominal lesions, macroscopically, the surface of the tumor was white-tending to brown, with varying amounts of hemorrhage and necrosis. Microscopically, the lesion displayed infiltrative hyperplasia in the abdominal adipose fibrous tissue (c and d, H&E, ×10). IHC showed moderate staining for CK (e ×40), MEL-CAM (f ×40), and HPL (h ×40), focally positive for HCG (g ×40). The Ki-67 proliferative index was about 20% (i ×40).

On 10th Jan. 2017, levels of t-HCG increased (6.17 IU/L) and subsequent abdominal ultrasound revealed two oval hypoechoic masses of sizes 17 × 5 mm and 8 × 5 mm, respectively, in subcutaneous soft tissues close to abdominal cesarean scar in our hospital. The two masses were approximately 8 mm and 4 m, respectively, away from the skin. The patient underwent her second time abdominal wall mass resection using ultrasound guidance (We cut apart skin and subcutaneous fat at the place 2 cm away from the original abdominal transverse incision). Macroscopically, the surface of the tumor was white-tending to brown, with varying amounts of hemorrhage and necrosis (Figures 1(a) and 1(b)). Microscopically, the lesion displayed infiltrative hyperplasia in the abdominal adipose fibrous tissue (Figures 1(c) and 1(d)). IHC showed moderate staining for CK (Figure 1(e)), MEL-CAM (Figure 1(f)), and HPL (Figure 1(h)), focally positive for HCG (Figure 1(g)). The Ki-67 proliferative index was about 20% (Figure 1(i)). These histological and immunohistochemical results were consistent with features of choriocarcinoma metastasis. Further chemo was not suggested after recurrence. Post operatively her serum t-HCG levels returned to normal. ETT was identified by pathological consultation with Sun Yat-sen University Cancer Center on 8th Feb.2017 after hematoxylin-eosin staining and immunohistochemical analysis, which showed that the tumor cells consisting of round or irregular atypical nuclei with abundant cytoplasm arranged in nests. Large scaled necrosis was discernible and nucleolus was occasionally observed in the atypical nuclei. Immunohistochemically, the tumor cells were strongly positive for CK, e-cadherin (E-cad), p40, p63, CD, EGFR, and p53. Tumor cells were focally positive for epithelial membrane antigen (EMA) and CK5/6, and positive for both HCG and HPL.

A diagnosis of ETT was also made by the Obstetrics & Gynecology Hospital of Fudan University after a biopsy curettage of the endometrium was done on 17th March 2017. The biopsy showed only endometrial hyperplasia without any endometrial evidence of ETT or CC.

The patient had been monitored at outpatient (serum t-HCG level, abdominal ultrasound, pelvic ultrasound, and chest X-ray) for 4 months until t-HCG increased to 7.20 mIU/mL in early July 2017. An ultrasound showed recurrence of an abdominal wall tumor of size 2.0 × 3.1 × 1.0 cm^3^ in the abdominal cesarean scar.

**Figure 2:**
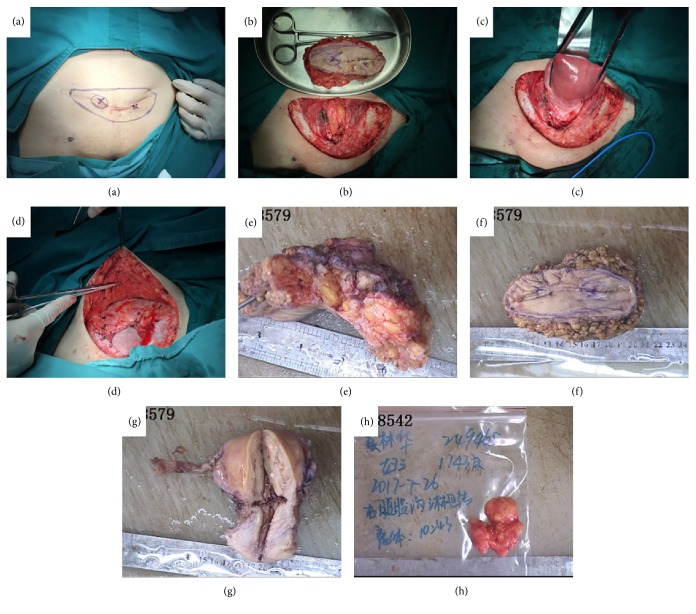
Abdominal hysterectomy, bilateral salpingectomy, left-sided ovarian cystectomy, the third time abdominal wall tumor resection and right-sided inguinal lymph node biopsy. (a, b, e, and f) The third time abdominal wall mass resectionthe tumor formed discrete nodules invading surrounding structures. (c and g) Abdominal hysterectomy specimens and bilateral salpingectomy. (h) Right-sided inguinal lymph node. (d) The repairing of abdominal wall defect.

Due to re-recurrence of the tumor, the following decisions have been made: total abdominal hysterectomy, bilateral salpingectomy, the third time abdominal mass resection (We cut apart skin and subcutaneous fat 3–4 cm away from the original abdominal transverse incision), right-sided inguinal lymph node biopsy and repair of abdominal wall defect (Figure 2). Her serum t-HCG level returned to normal after surgery.

The uterus, bilateral fallopian tubes, left-sided ovarian cyst and right-sided inguinal lymph node had no sign of metastasis or malignancy, while confirming isolated ETT and CC to the scar (abdominal wall tumor).

**Figure 3:**
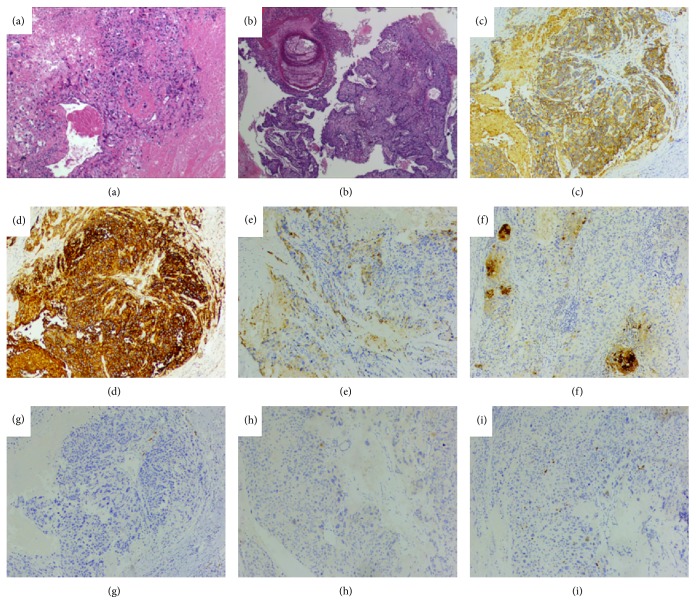
Histological feature of the third time abdominal wall tumor resection. Histologically, the tumor infiltrated adipose and fibrous tissue, and was surrounded by necrosis in the abdominal wall (a and b, H&E;×10). Scant syncytiotrophoblasts were immunoreactive to sall-4 (i ×40). The tumor cells were positive for E-Ca (c ×40), EGFR(d ×40). Moreover, EMA (e ×40) and HCG (f ×40) were both positive, and the cells were negative to HPL (g ×40) and PLAP (h ×40).

**Figure 4:**
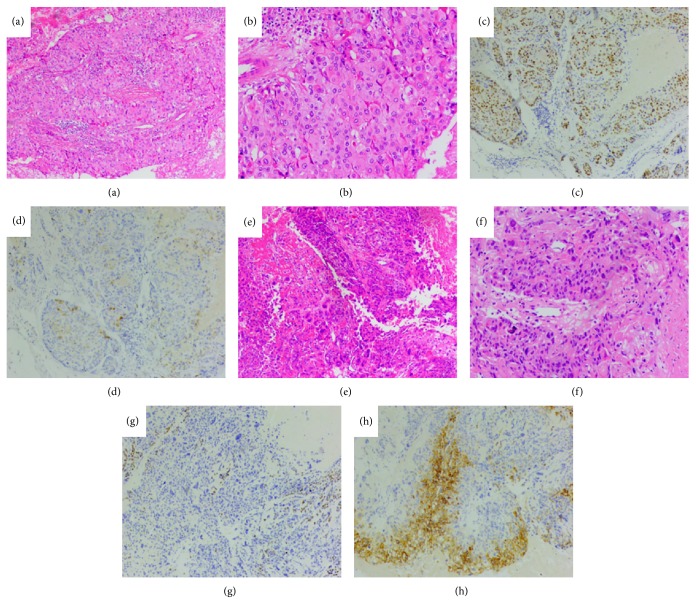
Features of epithelioid trophoblastic tumor and choriocarcinoma in the third time abdominal wall tumor resection. Most of the tumor tissue (ETT) (a ×40; b ×100) was characterized by nodular growth of medium-sized rumor cells arranged in nests or cords to large masses. The cells were relatively uniform. Nuclear atypia was generally moderate. Extensive necrosis appeared often. Small part of tumor tissue (CC) displayed a dimorphic population of trophoblast cells (syncytiotroblastic cells and cytotrophoblastic or intermediate trophoblastic cells) with marked hemorrhage and necrosis(e ×40; f ×100). Immunohistochemically, tumor cells were strongly positive for p63 (c ×40) and negative for inhibin-alpha (d ×40) in ETT, while they reacted positively to inhibin-alpha (h ×40 ) but negatively to p63 (g ×40) in CC.

Microscopically, the tumor infiltrated adipose and fibrous tissue and was surrounded by necrosis in the abdominal wall (Figure 3). Most of the tumor tissue (ETT) was characterized by nodular growth of medium-sized tumor cells arranged in nests or cords to large masses. The cells were relatively uniform. Nuclear atypia was generally moderate, Extensive necrosis appeared often. Small part of tumor tissue (CC) displayed a dimorphic population of trophoblast cells (syncytiotroblastic cells and cytotrophoblastic or intermediate trophoblastic cells) with marked hemorrhage and necrosis (Figure 4). Immunohistochemically, scant syncytiotrophoblasts were immunoreactive to sall-4. The tumor cells were positive for E-cad, EGFR. Moreover, EMA and HCG were both positive, but the cells were negative to HPL and placental alkaline phosphatase (PLAP) (Figure 3). Moreover, tumor cells were strongly positive for p63 and negative for inhibin-alpha in ETT, while they reacted positively to inhibin-alpha but negatively to p63 in CC (Figure 4). Combining with the patient's clinical presentation a final diagnosis of mixed GTN located in the cesarean scar was made (ETT 90% and CC 10%).

Currently, the patient is receiving outpatient follow-up measurements, including serum t-HCG level, abdominal ultrasound, pelvic ultrasound, and chest X-ray. The frequency of these follow-up measurements decreases as time goes on. The follow-up measurements started after the third time local tumor resection. In the first six months, measurements will be given weekly; then every three months since the seventh month and lasts for one year; then every half year and lasts for five years; then yearly for lifetime. This patient cooperates very well. At the time of reporting, no sign of recurrence. Follow-up is going on.

## 3. Discussion

Our case showed features of ETT and CC, so we regarded it as a mixed GTN. GTN, including both CC and ETT, is rarely seen in the abdominal wall, as it mostly presents in parts of the uterus [1]. However in our case, the uterus was negative for malignancy and the tumor was isolated to the abdominal wall. This patient had undergone two previous cesarean deliveries. In my viewpoint, this may mean metastasis from a primary uterine tumor that had regressed, or de novo transformation of trophoblastic cells.

In 2015, Zhang et al. [5] wrote a review and found 9 cases of mixed GTN in various combinations of ETT, CC, and PSTT. In addition, a report by Imamura in the same year describes a CC and ETT tumor of the uterine horn [8]. In mixed GTN, choriocarcinoma appears to be more common with either ETT or PSTT also present [5, 8].

The most common symptom of mixed GTN is abnormal vaginal bleeding, with possible increase of serum t-HCG levels [5]. The patient in our case presented atypically. She had no vaginal bleeding, and although, her t-HCG level increased, it was not as high as levels described in most cases of mixed GTN [5, 8]. This atypical presentation may be due to no involvement of endometrium and small size of the tumor. However, the tumor had the typical morphologic and immunophenotypic features of a mixed ETT and CC, focally immunoreactive to HPL, HCG, CK, and inhibin-alpha, and could be differentiated from PSTT by positive p63 immunostaining.

Low-risk GTN (FIGO Stages I–III: score <7) is treated with single-agent chemotherapy; High-risk GTN (FIGO Stages II–III: score <7 and Stage IV) is treated with multiple agent chemotherapy [9]. In principle, systemic therapies for metastatic intermediate trophoblastic tumor confirmed ETT include hysterectomy, excision of metastatic disease if feasible, and chemotherapy with a platinum/etoposide-containing regimen, such as EMA/EP, EP/EMA, or other regimens such as TP/TE, BEP, VIP, or ICE [9].

Therapeutic schema for mixed GTN is based on the differentiation of the neoplastic trophoblasts. Surgery is the primary treatment for mixed GTN [5]. The patient had abdominal mass resected twice. Due to re-recurrence of the tumor, the following decisions had been made: total abdominal hysterectomy, bilater salpingectomy, right-hand side inguinal lymph node biopsy, and a third time abdominal mass resection. We wanted to confirm that this was a recurrent GTN, and the elements of each recurrence are ETT and CC. Thus, we investigated and described in detail the excision specimens of the tumor in all three resections. We found that this tumor consisted of typical morphologic and immunophenotypic features of ETT and choriocarcinoma. Furthermore, we found that ETT was predominant and CC only focally.

All other specimens (uterus, fallopian tubes, ovarian cyst, and inguinal lymph nodes) were benign. With hindsight, we think resection of uterus, fallopian tubes, ovarian cyst, inguinal lymph nodes are not the appropriate management, because all specimens (uterus, fallopian tubes, ovarian cyst, inguinal lymph nodes) were benign and twice endometrial curettage demonstrated endometrial hyperplasia. But abdominal scar mass re-resection may be the appropriate management. The extent of abdominal scar mass re-resection was larger in the third time and at the time of reporting, the patient was negative for recurrence. So the extent of abdominal scar mass re-resection may be a successful management for this case.

The pathogenesis of ETT and CC remains unclear. In a model, proposed by Shih and Kurman, all three GTN (ETT, CC, and PSTT) are derived from trophoblastic stem cells. However, their levels of differentiation are different, this explains how they can coexist [8, 10]. Immature cells being more rapidly dividing are more sensitive to chemotherapeutic agents. This may explain why the CC is sensitive to chemotherapy while ETT and PSTT are not. Like pure ETT, ETT that coexists with CC is not responsive to the chemotherapy agents used in the treatment of CC. Chemotherapy is just an auxiliary therapy for ETT [9], platinum and etoposide (PE regimen) were applied to this patient after the first time mass resection. We did not use the more conventional chemotherapy approach of EP/EMA or TP/TE, because feasibility and effectiveness of PE regimen are not significantly different from those of the conventional chemotherapy approach, while the side effects of PE regimen are relatively lower [11–13].

The tumor recurred despite the patient had chemotherapy. Based on my study, not only because of chemotherapy insensitivity, but also the difficulty for chemotherapeutic drugs to reach the abdominal wall. So the patient did not receive the second time chemotherapy.

We report this case of coexisting ETT and CC next to the cesarean scar and in the subcutaneous tissues. In addition, detailed histopathologic examination with cytological analysis may be a diagnostic aide. Due to the rare presentation of this neoplasm, our understanding of its long-term prognosis and better treatments are limited. Therefore, long-term epidemiologic studies are necessary for better understanding of this neoplasm.
